# Response to Commentary on “Sedentary Leisure Behaviour, Physical Activity, and Gastroesophageal Reflux Disease: Evidence From a Mendelian Randomization Analysis”

**DOI:** 10.1002/hsr2.70933

**Published:** 2025-06-30

**Authors:** Shan Lu, Xingang Li, Ying Song

**Affiliations:** ^1^ Gastroenteric Medicine and Digestive Endoscopy Center The Second Hospital of Jilin University Changchun Jilin China; ^2^ School of Medical and Health Sciences Edith Cowan University Joondalup Western Australia Australia

Dear Editor,

We sincerely thank the authors of the letter entitled “Sedentary lifestyle, physical activity and healthy digital media use of Filipino adolescents: Review and policy insights” for their thoughtful and timely commentary on our recent publication [1]. Their insights highlight a critical and underexplored dimension of the sedentary behavior–health nexus, the patterns and determinants of lifestyle behaviors among adolescents in a digital age.

As the authors rightfully emphasize, Filipino adolescents represent one of the most sedentary youth populations globally, with a staggering 93.4% reported as physically inactive according to WHO estimates. The combination of rising screen‐based behaviors and low physical activity participation presents a pressing public health challenge, especially for children and adolescents [2]. We commend the authors for drawing attention to this demographic and advocating for integrative and multisectoral strategies that go beyond mere policy existence to ensure effective implementation, monitoring, and behavioral change.

Our study employed Mendelian randomization (MR) approaches [3] using large‐scale, adult‐based genome‐wide association studies (GWAS) to evaluate the potential causal links between leisure sedentary behaviors, physical activity, and gastroesophageal reflux disease (GERD). While this genetic approach provides robust evidence of directionality and minimizes confounding and reverse causation, we acknowledge that our findings primarily reflect adult populations of European ancestry. As such, they may not fully capture the unique behavioral, cultural, and developmental contexts of adolescents, particularly in countries with a younger population age structure, such as the Philippines.

In this regard, real‐world, adolescent‐focused research is crucial for translating genetic insights into age‐ and culturally appropriate interventions. The incorporation of national surveys such as the *Philippine Report Card on Physical Activity* and behavioral surveillance data [4], paired with implementation science and digital health innovation, can provide the granularity and context needed to inform policies that resonate with young people's realities.

We are especially encouraged by the call from the letter for a balanced digital media strategy that reduces sedentary screen time while harnessing technology for health promotion. This approach aligns well with emerging trends in digital public health and youth engagement, and we believe it holds promise for shaping positive behavioral trajectories early in life. We look forward to future studies that integrate genetic epidemiology with real‐world adolescent health data across diverse populations. Such work is essential to building a global evidence base that informs targeted, equitable, and sustainable public health strategies.

In conclusion, we thank the authors for extending the dialogue initiated by our MR study and for highlighting the urgent need for adolescent‐centered, culturally relevant health promotion efforts in the Philippines and beyond.

## Author Contributions

**Shan Lu:** writing – original draft. **Xingang Li:** writing – review and editing, conceptualization. **Ying Song:** writing – review and editing, conceptualization.

## Conflicts of Interest

The authors declare no conflicts of interest.

## Transparency Statement

The lead authors, Xingang Li and Ying Song, affirm that this manuscript is an honest, accurate, and transparent account of the study being reported; that no important aspects of the study have been omitted; and that any discrepancies from the study as originally planned are not applicable.

## Data Availability

Data sharing is not applicable to this article as no new data were created or analyzed in this study.
